# *Halyomorpha halys* (Hemiptera: Pentatomidae) Genetic Diversity in North America and Europe

**DOI:** 10.3390/insects10060174

**Published:** 2019-06-17

**Authors:** Despoina Ev. Kapantaidaki, Vassiliki I. Evangelou, William R. Morrison, Tracy C. Leskey, Jacques Brodeur, Panagiotis Milonas

**Affiliations:** 1 Department of Entomology and Agricultural Zoology, Benaki Phytopathological Institute, 8 St. Delta str., 14561 Kifissia, Greece; d.kapantaidaki@bpi.gr (D.E.K.); v.evangelou@bpi.gr (V.I.E.); 2 Center for Grain and Animal Health Research, USDA-ARS, 1515 College Ave., Manhattan, KS 66502, USA; William.Morrison@ARS.USDA.GOV; 3 Appalachian Fruit Research Station, USDA-ARS, 2217 Wiltshire Rd., Kearneysville, WV 25430, USA; tracy.leskey@ars.usda.gov; 4 Département de Sciences Biologiques, Institut de Recherche en Biologie Végétale, Université de Montréal, 4101 Rue Sherbrooke Est, Montréal, QC H1X 2B2, Canada; jacques.brodeur@umontreal.ca

**Keywords:** brown marmorated stink bug, haplotype diversity, population genetics

## Abstract

The brown marmorated stink bug, *Halyomorpha halys* (Hemiptera: Pentatomidae), is an invasive species in North America and Europe that damages many different host plants. Substantial work has been conducted on the genetic diversity and invasion pathways of *H. halys* in some of the countries where it has been found, based on mitochondrial sequences. The main objective of the present study was to further explore the genetic diversity of invasive populations of *H. halys* exploiting both mitochondrial and nuclear markers. We used two molecular markers: the mitochondrial Cytochrome Oxidase I (*COI*) gene, an ideal standardized molecular marker for distinguishing closely related species, and the ribosomal Internal Transcribed Spacer 1 (ITS1), because only a few sequences of *H. halys* exist to this point in global databases. We used specimens from eight populations from Greece, Italy, Canada, and the US. Among the 14 haplotypes retrieved based on the *mtCOI* gene, two of them (H162–H163) were detected for the first time. These two haplotypes were found in specimens from Canada, Italy, and the US. Concerning the ITS1 region, 24 haplotypes were identified, with 15 being unique for a sampled population. In Greece and the US, 14 and 12 haplotypes were found, respectively, with 7 and 6 of them being unique for Greece and the US, respectively. Our analysis of the nuclear genes of *H. halys* indicates high genetic diversity of the invading populations in North America and Europe.

## 1. Introduction

The brown marmorated stink bug, *Halyomorpha halys* (Stål) (Hemiptera: Pentatomidae), is native to China, Japan, Korea, and Taiwan, and has recently become a global invasive species [1,2,3]. It was likely first found outside of its native range in Allentown, Pennsylvania in 1996 [4], and confirmed in 2001 in the United States [4] and subsequently in Canada [5], Europe [6,7,8,9,10,11,12,13,14,15,16,17,18,19], Russia, Abkhazia, Georgia [20], and South America [21]. Based on recent modelling approaches, as well as newly reported distribution points, its dispersal will likely continue in the coming years. The brown marmorated stink bug is considered a major biosecurity concern for Australia and New Zealand [22,23,24]. In the new regions of its introduction in North America and Europe, it has become a devastating pest, causing significant damage and economic loss, as well as creating nuisance to residents in rural and urban areas [25]. *Halyomorpha halys* is an extremely polyphagous species, with more than 170 documented host plants, including many important agricultural crops [3,25].

The introduction and rapid spread of *H. halys* outside of its native range has elicited considerable efforts to identify the invasion pathways based on haplotype identification through sequencing. In North America, a single introduction from Beijing, China is most likely to have been the source of invasion of *H. halys* in the United States, which later spread to Canada. However, in Europe, multiple introductions have occurred, both from *H. halys* in its native range and possibly also from North America [6,14,26,27,28].

The genetic studies that have been conducted to elucidate the invasion history of *H. halys* in North America and Europe have focused mainly on the use of the mitochondrial Cytochrome Oxidase I (*COI*) gene, coupled with the Cytochrome Oxidase II (*COII*) gene [6,27,28], the 12S ribosomal RNA gene (12S *rRNA*) [28], and the cytochrome b (*cytb*) gene [14]. These studies have shown that there is considerable genetic diversity in the populations of *H. halys* in Europe, whereas the genetic variability in populations from North America is limited [14,26,28]. Further published studies reported secondary invasions, as well as continuous introductions into Europe and North America of the insect from its native range [27]. Although the *COI* gene has been extensively studied to elucidate the source and genetic variability of *H. halys* populations, other genetic markers, such as nuclear ones, have been neglected. To our knowledge, two studies based on sequences of the *28S* [14] and the Internal Transcribed Spacer 1 (ITS1) [29] have been conducted. No sequence variation was observed for the 28S D2 region in specimens collected in Japan, Korea, North America, and Europe (Switzerland), whereas eight unique haplotypes were obtained from 40 specimens from China, Korea, and Taiwan for a 416 bp fragment of the ITS1 region.

The ITS1 has been used for population genetic studies [30,31], and insects with a high level of polymorphism in the nuclear DNA appear to have high genetic diversity in the mitochondrial DNA as well [32]. The main objective of the present study was to further explore the genetic diversity of invasive populations of *H. halys* exploiting both mitochondrial and nuclear markers. Therefore, in the present study, we used two molecular markers. Firstly, the *COI* mitochondrial gene is an ideal standardized molecular marker for distinguishing closely related species, because it is characterized by a fast mutation rate and its sequence is conserved among conspecifics [33]. Secondly, the ITS1 nuclear region shows potential, but has been largely unexplored, since only a few sequences of *H. halys* exist in global databases (e.g., NCBI) [34].

## 2. Materials and Methods

### 2.1. Insect Material

We sampled and analysed eight populations of *H. halys* coming from Europe (Greece, Italy) and North America (USA, Canada) (Table 1). The populations from Athens, Greece, and Maryland, USA have been collected for a previous study [35]. The number of specimens for each country ranged between 1 and 50 (Table 1). Collected adults or nymphs were examined under a binocular stereoscope for species identification, and were preserved in 98% ethanol at −20 °C until used for molecular analysis.

### 2.2. DNA Isolation, Amplification, and Sequencing

The genomic DNA (gDNA) was isolated from a single leg from each specimen using a cetyltrimethyl ammonium bromide (CTAB)-based protocol, as described by Milligan [36]. The purity and quality of the extracted gDNA from each specimen was assessed using a nanophotometer (i.e., IMPLEN), and it was used as a template for the amplification of a partial fragment of the *COI* mitochondrial gene and a partial fragment of the ITS1 nuclear region. The primer pairs LCO—1490 (5′-GGTCAACAAATCATAAAGATATTGG-3′) and HCO—2198 (5′-TAAACTTCAGGGTGACCAAAAAATCA-3′) [37] were used for the amplification of a 658 bp fragment of the *COI* gene and the universal invertebrate primers, BD1 (5′-GTGTCCCATTTAATTAGTAGAGA-3′) and 4S (5′-TCTAGATGCGTTCGAAGTGTCGATG-3′) [34,38,39] for a 389–702 bp fragment of the ITS1 region.

Two microliters of the genomic DNA extract were used in 20 μL reactions consisting of 0.2 mM dNTPs, 1.0 μM of each primer, 1 μL Kapa Taq DNA polymerase (KapaBiosystems, Cape Town, South Africa), and 1× enzyme buffer (KapaBiosystems). Reactions for the *COI* gene were performed using the following profile: initial denaturation at 95 °C for 3 min, followed by five cycles of 95 °C for 1 min, 45 °C for 1 min, and 72 °C for 1 min, and by 35 cycles at 95 °C for 1 min, 50 °C for 1 min, and 72 °C for 1 min, and a final step of extension at 72 °C for 2 min. Reactions for the ITS1 region were performed using the following conditions: denaturating at 95 °C for 3 min, followed by 35 cycles of 95 °C for 30 s, 53 °C for 1 min, 72 °C for 1 min, and a final extension at 72 °C for 2 min. All reactions were run on a Verity 96-well Thermal Cycler (Applied Biosystems, Foster City, CA, USA).

Resulting PCR products were purified using the Nucleovac 96 Vacuum Manifold Kit (Macherey-Nagel, Düren, Germany) according to the manufacturer’s instructions, and sequenced in both directions (Macrogen Inc., Amsterdam, The Netherlands) using the same primers as in the PCR amplification.

### 2.3. Phylogenetic Analysis

Both forward and reverse partial *COI* and ITS1 sequences were obtained from a total of 59 and 100 specimens, respectively (Table 1). Consensus sequences were aligned using the multiple alignment algorithm MUSCLE in the software package Geneious Prime 19.1.1 [40] with default parameters. The same allele number was given for the identical sequences at each locus. The whole genome (Assembly Accession No.: GCA_000696795), including the complete mitochondrial genome (RefSeq ID: NC_013272), of *H. halys* is already available in NCBI [41]. Comparisons between the obtained sequences and those already published in previous studies [6,14,26,27,28,34] were performed with the use of the BLAST algorithm of NCBI (https://blast.ncbi.nlm.nih.gov/). Single sequences of each new haplotype were deposited in GenBank under the Accession Numbers MK779996 to MK779997 for *COI* and MK779975 to MK779995 for ITS1.

Aligned sequences were used for determining the phylogenetic relationships between specimens with the MEGA X version 10.0 [42] by using the Maximum Likelihood (ML) tree build method for each gene, respectively. The General Time Reversible model was chosen as the most appropriate one. To assess branch support, 1000 bootstrap replicates, as implemented in MEGA, were done for every analysis. Branches corresponding to partitions reproduced in less than 50% of bootstrap replicates were collapsed [43]. A discrete Gamma distribution was used to model evolutionary rate differences among sites (5 categories, +*G*, parameter = 0.3004 and 0.7690 for *COI* and ITS1, respectively). Published sequences for both genes available in the database were included as references in the phylogenetic analysis.

To produce diversity measures, the different sequences from the amplified fragment of the ITS1, generated from the 100 specimens of *H. halys*, were grouped according to the geographic collection area (Greece, Italy, Canada, USA) because amplification of ITS1 was successful only for a few individuals for the population from North Carolina, US. Diversity measures of the datasets for each country, namely the number of specimens (N), the number of sequences (n), the number of haplotypes (h) and unique haplotypes (H), haplotype diversity (Hd), nucleotide diversity per site (Pi), the number of variable polymorphic sites (S), the average number of nucleotide differences (k), the singleton variable (SVS) and parsimony informative sites (PIS), as well as the population genetic test, Tajima’s D, were calculated using the DNaSP v.6.12.01 package [44].

## 3. Results

### 3.1. Analysis of Cytochrome Oxidase I Gene

**Figure 1:**
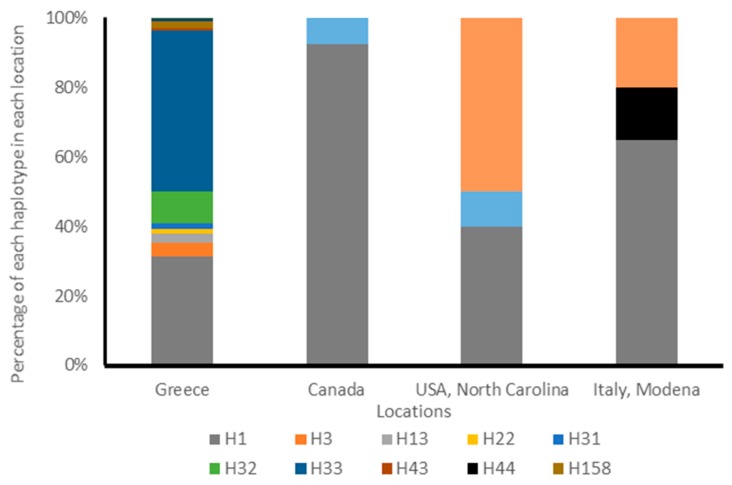
Summary of haplotype frequency of *Halyomorpha halys* adults based on the mitochondrial Cytochrome Oxidase I (*COI*) gene. The notation for the haplotypes is in accordance with data previously published in the literature.

Sequences of the *COI* gene fragment were analysed for a total of 59 specimens of *H. halys* that were collected from Greece, Italy, Canada, and the US. Among the 14 different haplotypes retrieved, two of them (H162–H163) were detected for the first time, and had not been previously described (Figure 1). These two haplotypes were found in specimens from Canada, Italy, and the US. The remaining haplotypes correspond to the available sequence data from previous studies [6,26,27,35]. The haplotype H1 was the most abundant and widespread, as it was shared among specimens from all four countries. It was not found in the specimens from both locations, Chania (Crete) and Peloponnese, Greece. The two specimens from Peloponnese belonged to H32 and the one from Crete to H33; those haplotypes have also been found in the population from Athens. The new haplotype H163 was commonly found (50%) in specimens from North Carolina, US (Figure 1).

**Figure 2:**
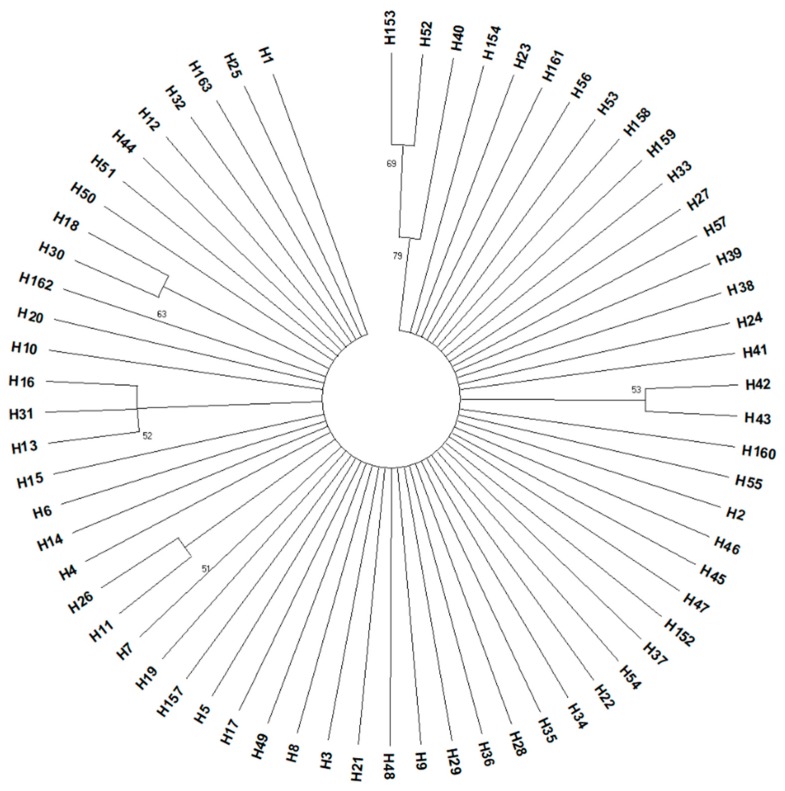
Phylogenetic tree constructed on haplotypes of *COI* for *Halyomorpha halys* using the Maximum Likelihood Tree Build Method based on the General Time Reversible model. Percentage bootstrap values from 1000 replicates superior to 50% are given at each node. Based on data from the present study and [6,14,26,27,35,45].

A phylogenetic tree was constructed from sequences of the *COI* gene using the new haplotypes detected in this study and the already identified haplotypes from previous studies existing in the National Center for Biotechnology Information, NCBI (https://www.ncbi.nlm.nih.gov/) (Figure 2). Based on their position in the phylogenetic tree, the new haplotypes detected in specimens from Italy, US, and Canada (H162 and H163) cluster with haplotypes being detected in other surveys conducted in China, Korea, US, Canada, and Europe [45].

### 3.2. Analysis of Internal Transcribed Spacer 1

**Figure 3:**
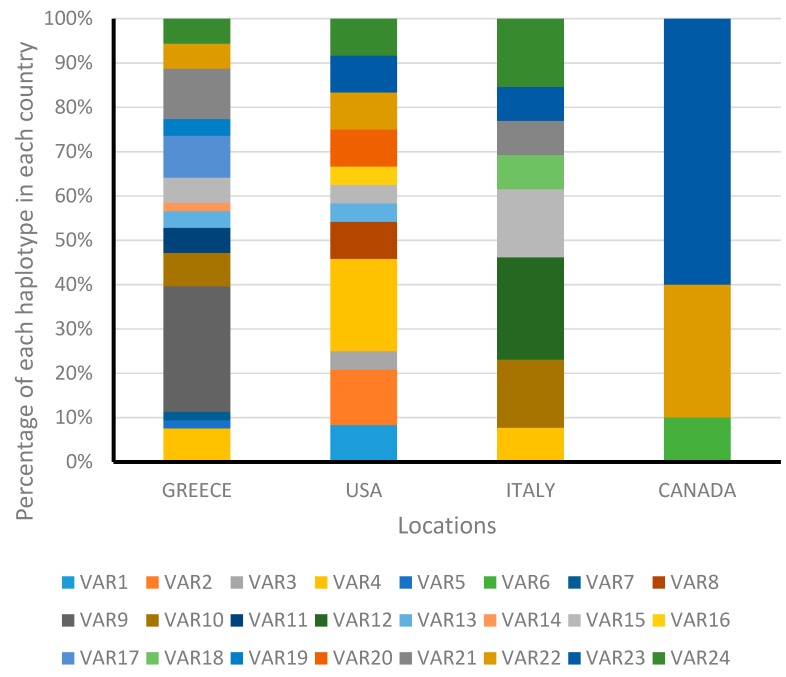
Summary of haplotype frequency of *Halyomorpha halys* adults based on Internal Transcribed Spacer 1 (ITS1) region.

**Figure 4:**
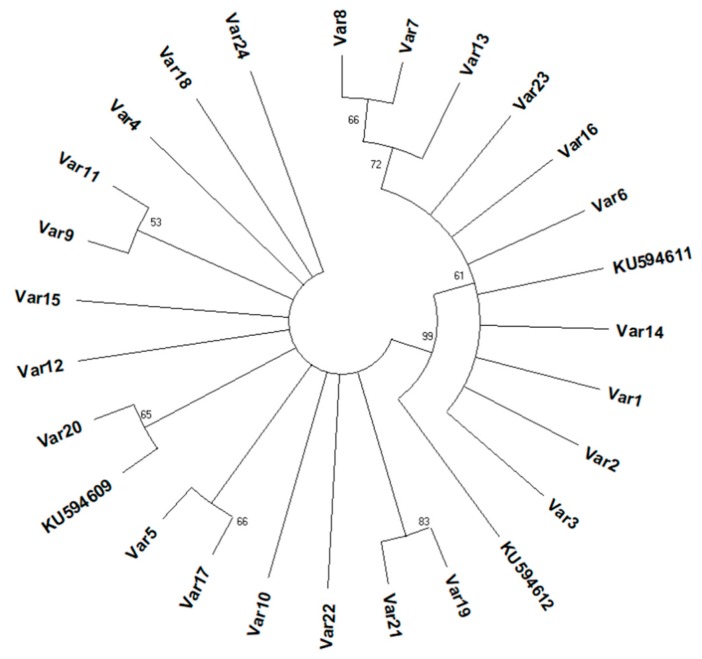
Phylogenetic tree constructed on Internal Transcribed Spacer 1 (ITS1) sequences for *Halyomorpha halys* using the Maximum Likelihood Tree Build Method, based on the General Time Reversible model. Percentage bootstrap values from 1000 replicates superior to 50% are given at each node. The ITS1 sequences from Valentin et al. (2015) are included [34].

In total, 100 individuals were sequenced for the ITS1 region, and 24 variants (denoted as VAR1-VAR24) were identified. The occurrence and frequency of each variant in each sampling site are shown in Figure 3. Among the 24 different variants, 15 occurred in just one population. In Greece and the US, 14 and 12 variants were found, respectively, with 7 and 6 of them being unique for Greece or the US. The populations from Italy and Canada included 8 and 3 variants, with 2 and 1 being unique, respectively, for each country (Table 2). The two most common variants in specimens collected from Greece were VAR9 (28.3%) and VAR21 (11.3%), followed by VAR17 (9.4%), VAR4, and VAR10 (each 7.5%). The population from Canada was predominated by VAR23 (60%). Specimens from Italy and the US were more evenly distributed among the different variants detected (Figure 3). Sequences of the ITS1 region from 100 specimens were used to construct a phylogenetic tree (Figure 4). The phylogenetic tree reveals structuring among the variants and divides the ribosomal DNA sequences of *H. halys* into two large clusters, both of which consisted of specimens collected from all four countries, without clustering specimens sampled from a single country into the same cluster. The first cluster was dominated by specimens from the US, and the second one by specimens from Greece. In addition, high divergence was observed in the US and Greece, since most of the variants in both countries were clustered into private lineages.

Genetic diversity data for ITS1, based on haplotype and nucleotide diversity are shown in Table 2. The specimens from Greece had the highest haplotype and nucleotide diversity, which were 0.830 ± 0.034 (SD) and 0.022 ± 0.001 (SD), respectively. The specimens from the US also had high haplotype and nucleotide diversity, and were found to be 0.728 ± 0.062 (SD) and 0.023 ± 0.012 (SD), respectively. The specimens from Italy and Canada had similar levels of haplotype and nucleotide diversity (Table 2). The polymorphic sites were 82, 64, 12, and 10 for Greece, the US, Canada, and Italy, respectively. Within these polymorphic sites, there were 80, 12, 9, and 2 parsimony informative sites, and 2, 52, 3, and 8 singleton variable sites, respectively. In Greece and the US, Tajima’s D values were found to be significantly negative, indicating a possible demographic expansion following a bottleneck event.

## 4. Discussion

Until recently, the genetic variation of *H. halys* in the US based on *COI* was considered very low [29]. Approximately 10 years after its introduction in the US, only two haplotypes have been detected. Recently, Valentin et al. [45] and Lee et al. [46] reported additional haplotypes from specimens collected in the US, both in Eastern and Western states. In this paper, we reported two novel haplotypes from the US, increasing the total number to seven. Although this number remains relatively smaller than for haplotypes detected in other invaded countries, the genetic diversity of *H. halys* in the US is not as low as was initially found [28]. It is likely that there had been a single event of introduction in the US which was followed by additional introductions from the native range of *H. halys*. However, we cannot exclude the possibility that the initial introduced population was dominated by the H1 haplotype, hampering the sampling and detection of other haplotypes.

Our study confirms higher COI haplotype diversity in Europe than in North America [14,26], with 13 and 3 haplotypes found in each continent, respectively. Despite extensive analyses of *H. halys* haplotype diversity in Europe (e.g., [6,14,26,27]), we have documented one new haplotype (also found in the US), suggesting ongoing invasion and re-introduction of *H. halys* in Europe.

This is the first study to report detailed genetic variability of *H. halys* based on the ITS1 region in countries outside its native range. The introduction and spread of *H. halys* in North America and Europe have triggered research efforts to identify the pathways of introduction based on the underlying genetics of populations originating from native and introduced areas [6,14,26,27,46]. Most of these studies performed genetic analyses on the mitochondrial *COI* gene. A few studies were performed on the genetic variability of *H. halys* by analyzing the *COII* gene [6,27,29], the 12S ribosomal RNA gene (12S *rRNA*) [29], and the cytochrome b (*cytb*) gene [14,29]. On the other hand, studies of genetic variability based on nuclear genes are limited [28]. Here, we reported genetic variability for *H. halys* based on sequences of the ITS1 region from 100 specimens. Only three sequences for ITS1 have been reported up to now in the literature [34]. In the present study, 24 new variants were identified from specimens originating from North America and Europe. The geographic distribution of variants shows that only VAR4 occurs in all countries, except for Canada. Among the other variants, several (VAR1, VAR2, VAR3, VAR8, VAR16, and VAR20) are unique for the US and Greece (VAR5, VAR7, VAR9, VAR11, VAR14, VAR17, and VAR19). Unique variants are also identified in specimens from Canada and Italy. It is important to note that the greater sample size for Greece (*n* = 53) likely permits a larger resolution of genetic diversity compared to the other countries examined, where sample sizes were much lower. Interestingly, the US was the second most diverse country.

While the low number of *COI* haplotypes historically found in the US was considered an indication of a single introduction, at least in the eastern part of the US, our combined analysis of *COI* and ITS has demonstrated a much higher genetic diversity from specimens in the US. This suggests multiple introductions of *H. halys* in the US, as also suggested for Europe. Although invasive species initially experience a decrease in genetic diversity, it has been suggested that high genetic diversity due to multiple introductions might allow a species to become invasive, and subsequently to establish, persist, and disperse into new habitats [47]. Genetic diversity enhances the capacity for adaptive evolution and population success, since heritable genetic variation is a prerequisite for adaptation to new conditions, which occurs in many introduced species [48]. The high genetic diversity of *H. halys* occurring both in Europe and North America indicates that the invasions are not affected by genetic bottlenecks or founder effects; two of the most dominant processes influencing the genetic diversity of a species in its new range [47].

## 5. Conclusions

In summary, our results demonstrate that by exploiting both mitochondrial and nuclear markers, we were able to identify substantial genetic diversity from specimens in the US. Detection of the source area of an invasion is more accurate when multiple molecular markers are combined. The greater diversity of the ITS1 region relative to the mitochondrial *COI* gene presents an interesting wrinkle in unraveling the population genetics of this important and devastating invasive species.

## Figures and Tables

**Table 1:** Populations of *H. halys* used in this study and total number of specimens from each population analyzed for the molecular markers Cytochrome Oxidase I (*COI*) and Internal Transcribed Spacer 1 (ITS1).

Country	Region	*COI*		ITS1	
		Morrison et al. 2017 [35]	This Study	Morrison et al. 2017 [35]	This Study
Greece	Athens	195		50	
	Peloponnese		2		2
	Chania		1		1
Italy	Modena		20		11
	Perugia		3		2
USA	Smithsburg, Maryland	60		19	
	North Carolina		20		5
Canada	London, Ontario		13		10

**Table 2:** Genetic diversity of *H. halys* populations from Canada, Greece, the US, and Italy, assessed using on Internal Transcribed Spacer 1 (ITS1) sequences. *N*, number of specimens; *n*, number of sequences; h, number of haplotypes; H, number of unique haplotypes; Hd, haplotype (gene) diversity; Pi, nucleotide diversity (per site); S, number of variable polymorphic sites; k, average number of nucleotide differences; SVS, singleton variable sites; PIS, parsimony informative sites.

Region	N	n	h	H	Hd ± SD	Pi ± SD	S	k	SVS	PIS	Tajima’s D	*p*-Value	Fu’s Fs
Canada	10	10	3	1	0.600 ± 0.131	0.011± 0.003	12	4.800	3	9	0.594	*p* > 0.10	4.717
Greece	53	53	14	7	0.830 ± 0.034	0.022 ± 0.001	82	7.896	2	80	−2.083	*p* < 0.05	3.967
USA	24	24	12	6	0.728 ± 0.062	0.023 ± 0.012	64	7.909	52	12	−2.138	*p* < 0.05	5.890
Italy	13	13	8	2	0.603 ± 0.131	0.005 ± 0.002	10	1.897	8	2	−1.643	*p* > 0.05	0.934
